# Visualization analysis of poisoning-related research based on CiteSpace

**DOI:** 10.3389/fpubh.2025.1592916

**Published:** 2025-05-07

**Authors:** Wen Zhu, Yao Xiao, LinShen Xie

**Affiliations:** West China School of Public Health and West China Fourth Hospital, Sichuan University, Chengdu, China

**Keywords:** CiteSpace, poisoning, visualization research, oxidative stress, gut microbiota

## Abstract

**Objective:**

Summarize the current status, hotspots, and frontier trends in poisoning research over the past decade using CiteSpace software, and provide direction for future research in toxicology and public health.

**Methods:**

Relevant literature published between 2015 and 2024 was retrieved from the China National Knowledge Infrastructure (CNKI) and Web of Science Core Collection. CiteSpace software was employed to conduct a visual analysis of poisoning-related research literature from the past decade.

**Results:**

A total of 5,644 Chinese articles and 14,985 English articles were included. High-frequency keywords in Chinese literature included “hemoperfusion” and “nursing” while high-frequency keywords in English literature included “exposure” “oxidative stress” and “identification.” Keywords with strong betweenness centrality included “treatment,” “blood purification” and “carbon monoxide.” Recent emerging hotspots in Chinese literature were “liver function” and “oxidative stress” while in English literature, “gut microbiota” has recently gained attention.

**Conclusion:**

Poisoning-related research has shown an overall upward trend. The research hotspots in this field primarily include epidemiology, oxidative stress, clinical treatment, prognosis, and chemical substances (e.g., CO, paraquat). In-depth studies on gut microbiota and oxidative stress are expected to become future research trends in this field.

## 1 Introduction

Poisoning is one of the significant causes of morbidity and mortality worldwide (1), with its etiology being complex and multifaceted, involving exposure to a variety of substances. According to *The Lancet* Global Burden of Disease Study (GBD 2019), poisoning caused hundreds of thousands of deaths worldwide in 2019 (including both intentional and unintentional poisoning), with developing countries accounting for over 70% of these fatalities (2). The global medical community places high importance on poisoning prevention and treatment. Leading authorities such as the American Association of Poison Control Centers (AAPCC) and the European Association of Poisons Centers and Clinical Toxicologists (EAPCCT) have established multiple clinical management guidelines, emphasizing the importance of early identification, precise intervention, and standardized treatment.

Substances that cause poisoning include pharmaceuticals [such as barbiturates (3)], chemicals (such as industrial chemicals, pesticides, and household cleaners) (4), as well as natural toxins derived from plants and animals (such as toxic mushrooms and snake venoms) (5). Based on the duration and nature of exposure, poisoning can be classified into acute poisoning (short-term exposure to high doses of toxic substances), chronic poisoning (long-term exposure to low doses of toxic substances), and subacute poisoning (repeated or continuous exposure to moderate doses of toxic substances over a relatively short period). The causes of poisoning may be intentional (such as suicide or poisoning) (6) or accidental (such as occupational exposure or ingestion) (7).

With the acceleration of industrialization, a large number of new chemicals have been widely applied in industrial production, agriculture, medicine, and daily life. While the extensive use of these chemicals has driven social progress, it has also significantly increased human exposure to toxic substances, posing potential health and environmental risks. For instance, heavy metal pollution in industrial wastewater, pesticide residues threatening food safety (8), and the improper use of chemicals in daily life can all serve as triggers for poisoning incidents. Currently, there is a lack of systematic bibliometric research in the field of poisoning on a global scale, resulting in an incomplete understanding of research trends, knowledge structures, and regional contributions in this domain. Although existing international consensus frameworks, such as the Globally Harmonized System of Classification and Labeling of Chemicals (GHS), provide a classification system for toxic substances, quantitative analysis of research trends remains insufficient. Therefore, it is necessary to utilize CiteSpace to conduct a visual analysis of research related to poisoning, in order to identify current research hotspots and frontier trends.

Bibliometrics is a method of objectively evaluating the development status and trends of a research field through quantitative analysis of literature. CiteSpace is a Java-based network application primarily used for data analysis and visualization research. As a representative tool in the field of information visualization analysis, its unique functionalities play an important role in academia (9).

This study innovatively employs bibliometric methods to conduct a comprehensive analysis of global poisoning-related research, systematically reviewing the current international research landscape while specifically exploring the contributions and evolving trends of Chinese-language literature in this field. This study employs CiteSpace 6.4.R1 software to conduct visual analysis of literature in the field of poisoning, aiming to comprehensively understand the research status of poisoning over the past decade, identify key research priorities and directions, capture frontier trends, and promote global academic exchange and collaboration in poisoning research.

## 2 Data and methods

### 2.1 Data sources

China National Knowledge Infrastructure (CNKI) is the most influential comprehensive academic resource platform in China. To ensure the comprehensiveness and representativeness of research data, we used the CNKI database as our retrieval source, with the search scope limited to academic journals. Advanced search was employed with all journal types selected, covering the time period from 2015 to 2024.

Web of Science (WoS) is one of the world’s most authoritative academic research platforms, encompassing high-quality literature resources from internationally renowned publishers. To ensure the authority and representativeness of the research data, this study selected the Web of Science Core Collection (WoSCC) as the primary data source, with the citation index set to Science Citation Index Expanded (SCI-Expanded). The exact match search option was enabled, covering the time period from 2015 to 2024.

To ensure comprehensive literature coverage, the search strategy was designed as follows: Chinese search formula: (poisoning OR Acute poisoning OR Chronic poisoning). English search formula: (poisoning OR toxicosis OR Acute poisoning OR Chronic poisoning OR Acute toxicosis OR Chronic toxicosis).

### 2.2 Inclusion and exclusion criteria

**Figure 1:**
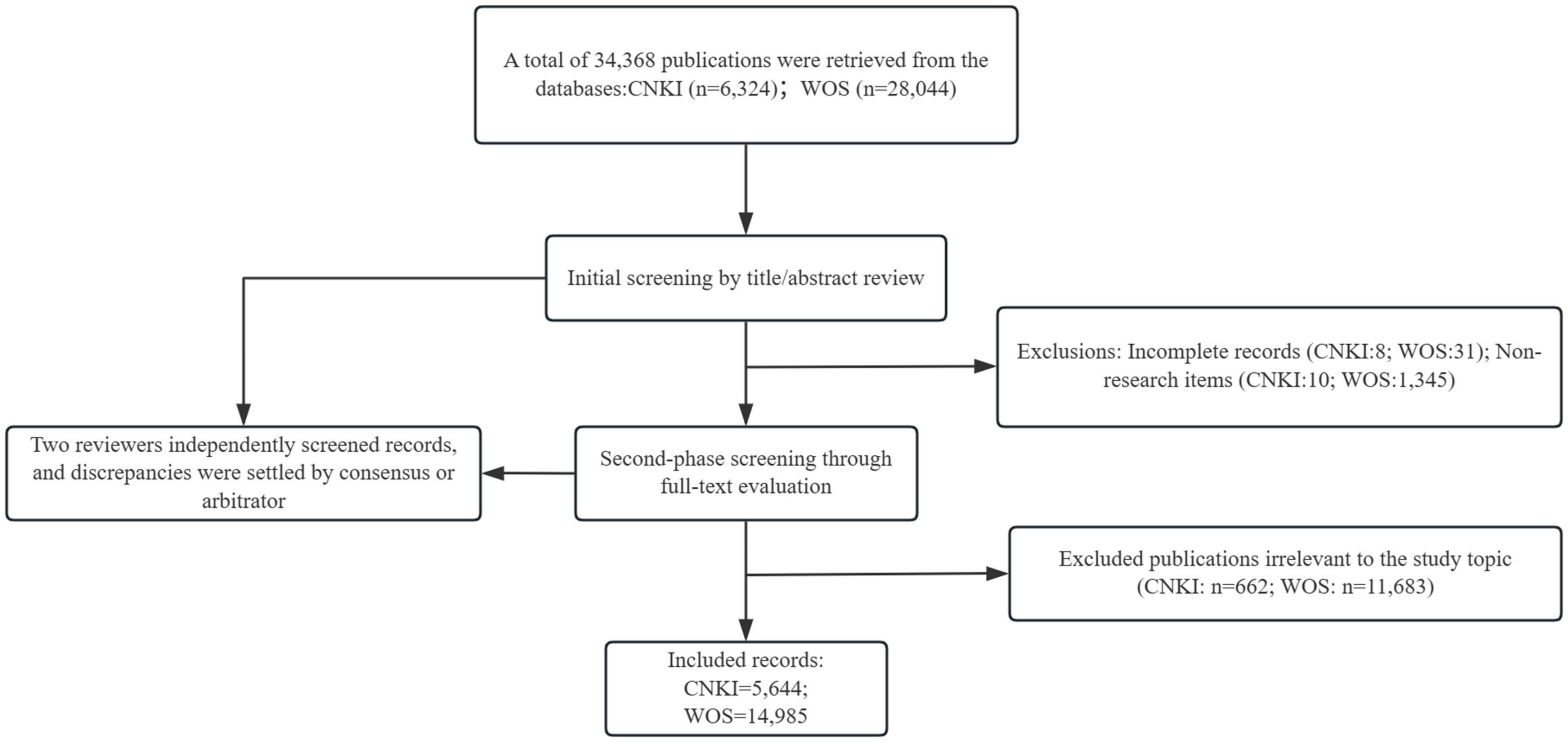
Literature screening and selection process flowchart.

The literature screening followed a two-stage process. First, articles were excluded based on titles/abstracts if irrelevant or incomplete. Second, full texts were assessed against predefined criteria. Two researchers independently conducted screening, with disagreements resolved through discussion or third-party arbitration, ensuring rigorous selection. Inclusion criteria: Academic journal literature focusing on poisoning published between 2015 and 2024. Exclusion criteria: (1) Literature without authors; (2) Incomplete information; (3) Invalid literature types such as conference papers, notices, news, speeches, etc.; (4) Literature irrelevant to the research topic (Figure 1).

### 2.3 Methods

The search results from the WOS database were exported in plain text format with “Full Record and Cited References,” while those from the CNKI database were exported in “Refworks” format and saved under the filename “download_.” The data were then analyzed using CiteSpace 6.4.R1. Parameter settings: The time span for both Chinese and English literature was set from 2015 to 2024, with time slices set to 1 year, threshold set to Top 50, and network pruning methods set to Pathfinder and Pruning sliced networks. All other settings were kept as system defaults. Conduct author analysis, institutional distribution mapping, country/region distribution analysis, keyword co-occurrence examination, clustering and burst detection analyses, and generate knowledge mapping visualizations.

## 3 Results

### 3.1 Annual publication volume

**Figure 2:**
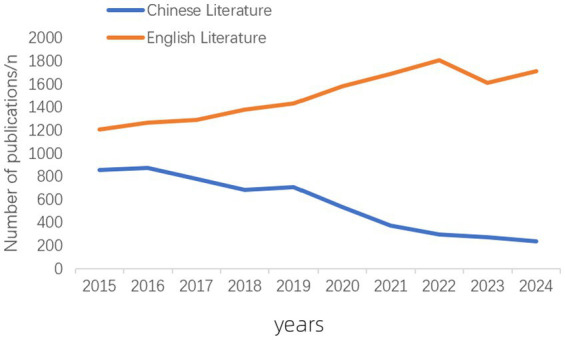
Annual publication volume of Chinese and English literature in poisoning research.

A total of 6,324 relevant articles were retrieved from the CNKI database, and 28,044 relevant articles were retrieved from the WOS database. After excluding non-compliant literature, 5,644 Chinese articles (effective literature inclusion rate: 89.24%) and 14,985 English articles (effective literature inclusion rate: 53.43%) were finally included. The number of English publications has gradually increased since 2015, reaching a peak in 2022, showing an overall upward trend. In contrast, the number of Chinese publications has gradually declined since 2015, reaching its lowest point in 2024, showing an overall downward trend. Additionally, the annual number of English publications consistently exceeded that of Chinese publications. This trend demonstrates the accelerating internationalization of poisoning research. The growth in English-language publications reflects dynamic global developments in toxicology, clinical poisoning treatment, and environmental toxin studies, with researchers increasingly favoring international journals to facilitate cross-border collaboration. The comprehensive coverage of English databases (e.g., Web of Science) and the dominant role of SCI/SSCI journals in research evaluation have further reinforced this trend (Figure 2).

### 3.2 Author analysis

The study incorporated a total of 684 authors from WOS and 525 authors from CNKI. In the author collaboration network visualization, each node represents an individual author, with node size and color saturation directly proportional to publication output. The connecting lines between nodes indicate collaborative relationships, where line thickness corresponds to collaboration strength and color denotes the year of cooperation. Clusters of densely interconnected nodes reveal established research teams within the network. This comprehensive visualization effectively demonstrates multidimensional research characteristics including individual productivity, collaboration intensity, and team structure.

**Figure 3:**
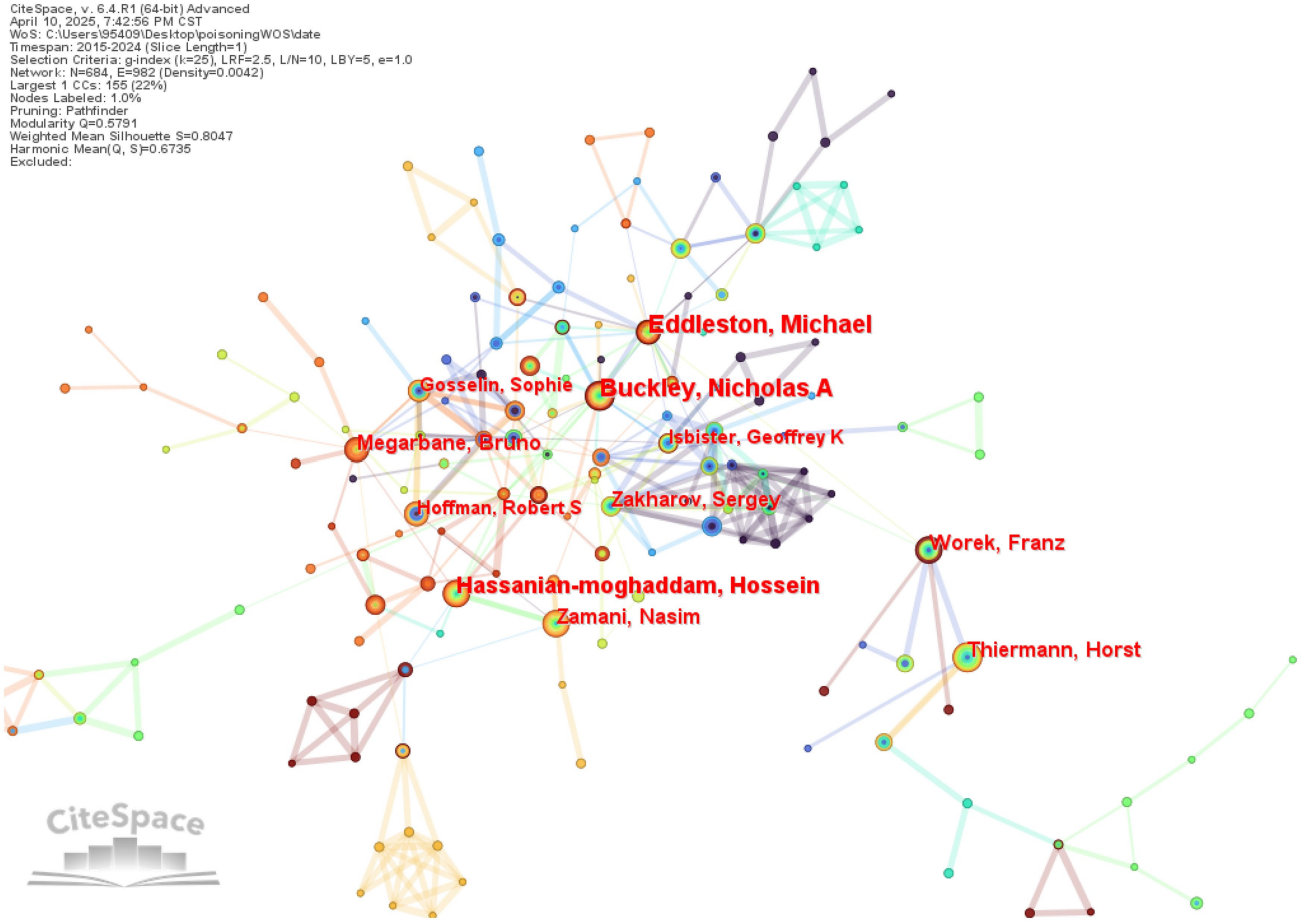
Co-authorship network of English literature authors.

**Figure 4:**
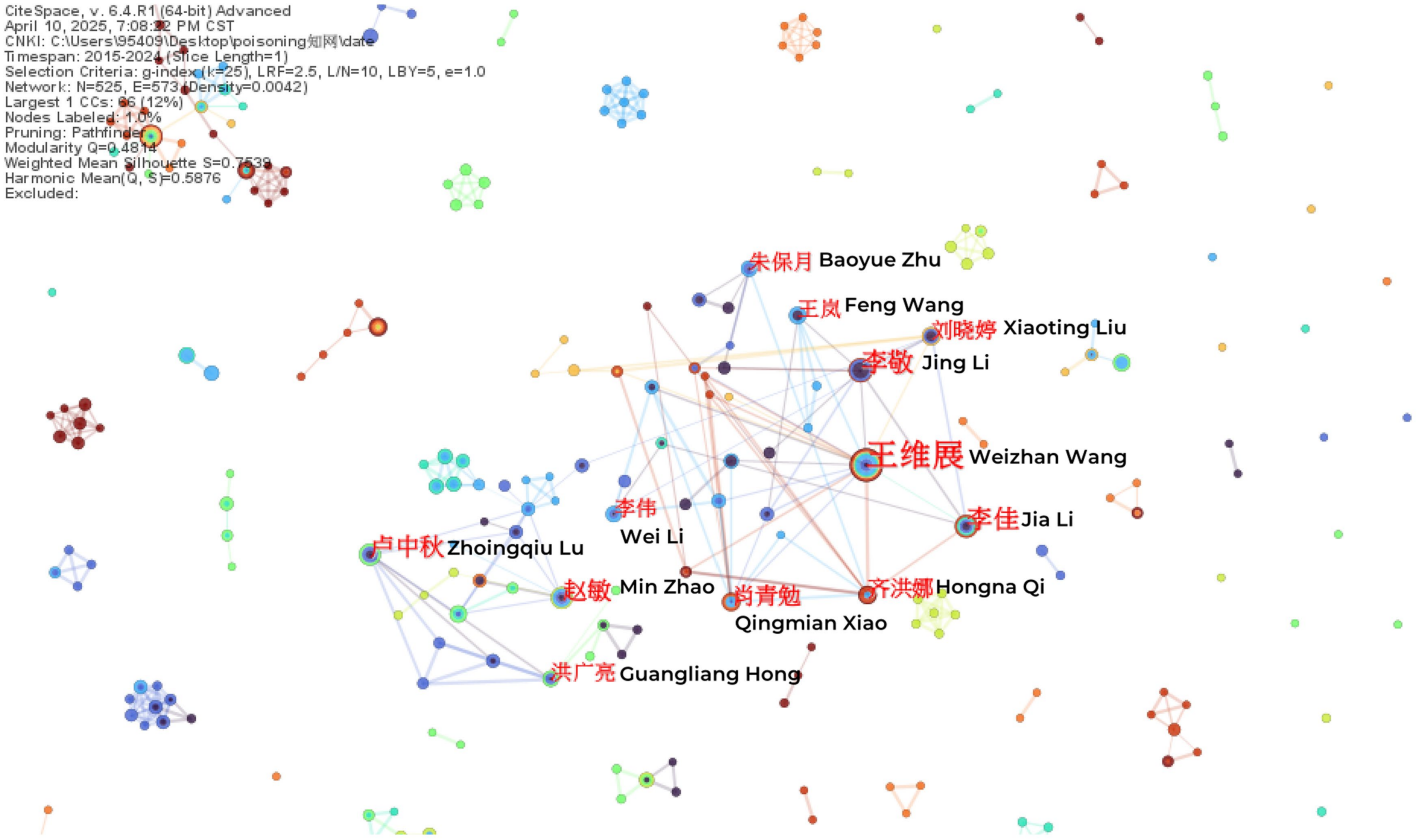
Co-authorship network of Chinese literature authors.

Authors with the highest publication output were identified as core researchers in this field. Table 1 presents the top 10 most prolific authors. The leading contributors in English literature were Buckley, Nicholas A (73 publications), Eddleston, Michael (71), and Riet-correa, Franklin (57), while Weizhan Wang (40 publications), Xiangdong Jian (28), and Jing Li (20) ranked highest in Chinese literature. Notably, Buckley, Nicholas A and Eddleston, Michael in the English dataset, along with Weizhan Wang and Zhongqiu Lu in the Chinese dataset, maintained extensive collaboration networks, each forming a distinct research cluster centered around their work (Figures 3, 4).

**Table 1:** Top 10 authors by publication volume in Chinese and English literature in toxicology research.

label	Author of English literature	Number of publications	Author of Chinese literature	Number of publications
1	Buckley, Nicholas A	73	Weizhan Wang	40
2	Eddleston, Michael	71	Xiangdong Jian	28
3	Riet-correa, Franklin	57	Jing Li	20
4	Hassanian-moghaddam, Hossein	55	Jia Li	19
5	Thiermann, Horst	54	Zhongqiu Lu	16
6	Zamani, Nasim	50	Wen Yi	16
7	Turner, Andrew D	47	Min Zhao	16
8	Worek, Franz	44	Yihong Yang	13
9	Megarbane, Bruno	44	Xin Wang	13
10	Zakharov, Sergey	38	Qingmian Xiao	13

### 3.3 Institutional analysis

**Figure 5:**
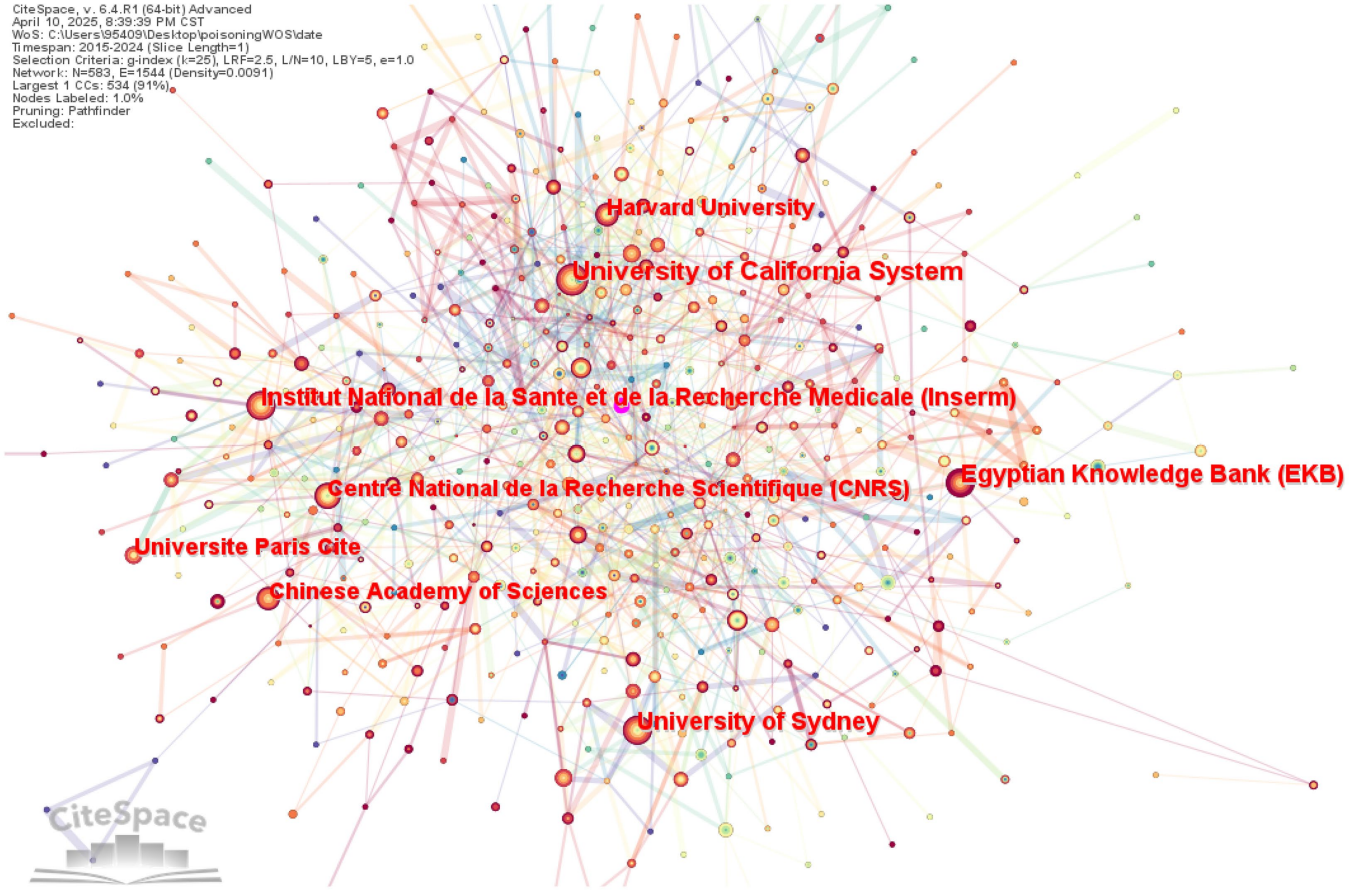
Institutional co-occurrence network in English literature.

The study conducted a visualization analysis with “institution” as the node type. Table 2 presents the top 10 most productive research institutions. In English literature, the University of California System leads poisoning research with 276 high-quality publications, followed closely by the Egyptian Knowledge Bank (EKB) (220 publications) and Institut National de la Santé et de la Recherche Médicale (Inserm) (217 publications), demonstrating their significant academic influence in this field (Figure 5).

**Table 2:** Top 10 institutions by publication volume in Chinese and English literature in toxicology research.

label	Institutions of English literature	Number of publications	Institutions of Chinese literature	Number of publications
1	University of California System	276	Emergency Department, Shengjing Hospital of China Medical University	23
2	Egyptian Knowledge Bank (EKB)	220	Shenyang Ninth People’s Hospital	14
3	Institut National de la Santé et de la Recherche Médicale (Inserm)	217	Emergency Department, Xijing Hospital of the Fourth Military Medical University	14
4	University of Sydney	194	National Institute of Occupational Health and Poison Control, Chinese Center for Disease Control and Prevention	12
5	Centre National de la Recherche Scientifique (CNRS)	166	Emergency Department, The First Affiliated Hospital of Henan University of Science and Technology	11
6	Chinese Academy of Sciences	158	Department of Poisoning and Occupational Diseases, Emergency Department, Qilu Hospital of Shandong University	11
7	Harvard University	150	Emergency Department, West China Hospital of Sichuan University	11
8	Universite Paris Cite	146	The First Affiliated Hospital of Henan University of Science and Technology	10
9	Assistance Publique Hopitaux Paris (APHP)	129	Emergency Department, The First Affiliated Hospital of China Medical University	10
10	University of New South Wales Sydney	129	Department of Poisoning and Occupational Diseases, Qilu Hospital of Shandong University	10

**Figure 6:**
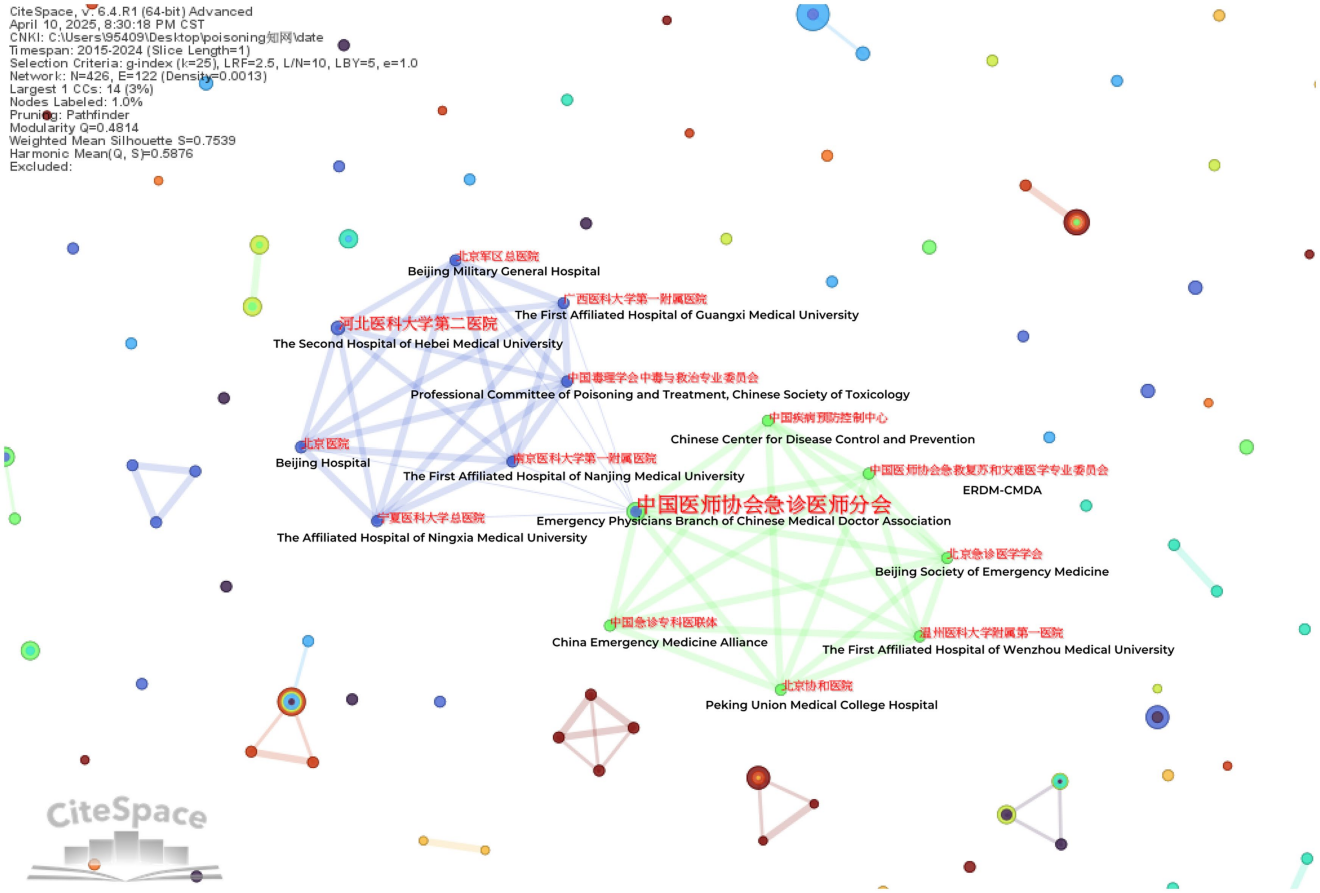
Institutional collaboration network in Chinese literature.

For Chinese literature, the Emergency Department at Shengjing Hospital of China Medical University ranked first with 23 publications. Notably, the Emergency Physicians Branch of the Chinese Medical Doctor Association serves as a central hub in the collaboration network, maintaining strong research partnerships with institutions including The First Affiliated Hospital of Nanjing Medical University and the Chinese Center for Disease Control and Prevention (Figure 6).

### 3.4 Country/region analysis

The bibliometric analysis based on the Web of Science (WOS) database revealed the top 10 most productive countries/regions in poisoning research, as presented in Table 3. China ranked first with 3,138 publications, followed by the United States (2,764) and Japan (771). Notably, China and the U.S. demonstrated a substantial lead in publication output, creating a significant gap with other countries. This prominent advantage strongly reflects both nations’ strategic emphasis and sustained investment in poisoning research.

**Table 3:** Top 10 countries by publication volume in English literature in toxicology research.

Label	Country of English literature	Number of publications
1	People’s Republic of China	3,138
2	USA	2,764
3	Japan	771
4	Australia	691
5	France	677
6	England	670
7	Germany	633
8	Brazil	597
9	India	565
10	South Korea	544

**Figure 7:**
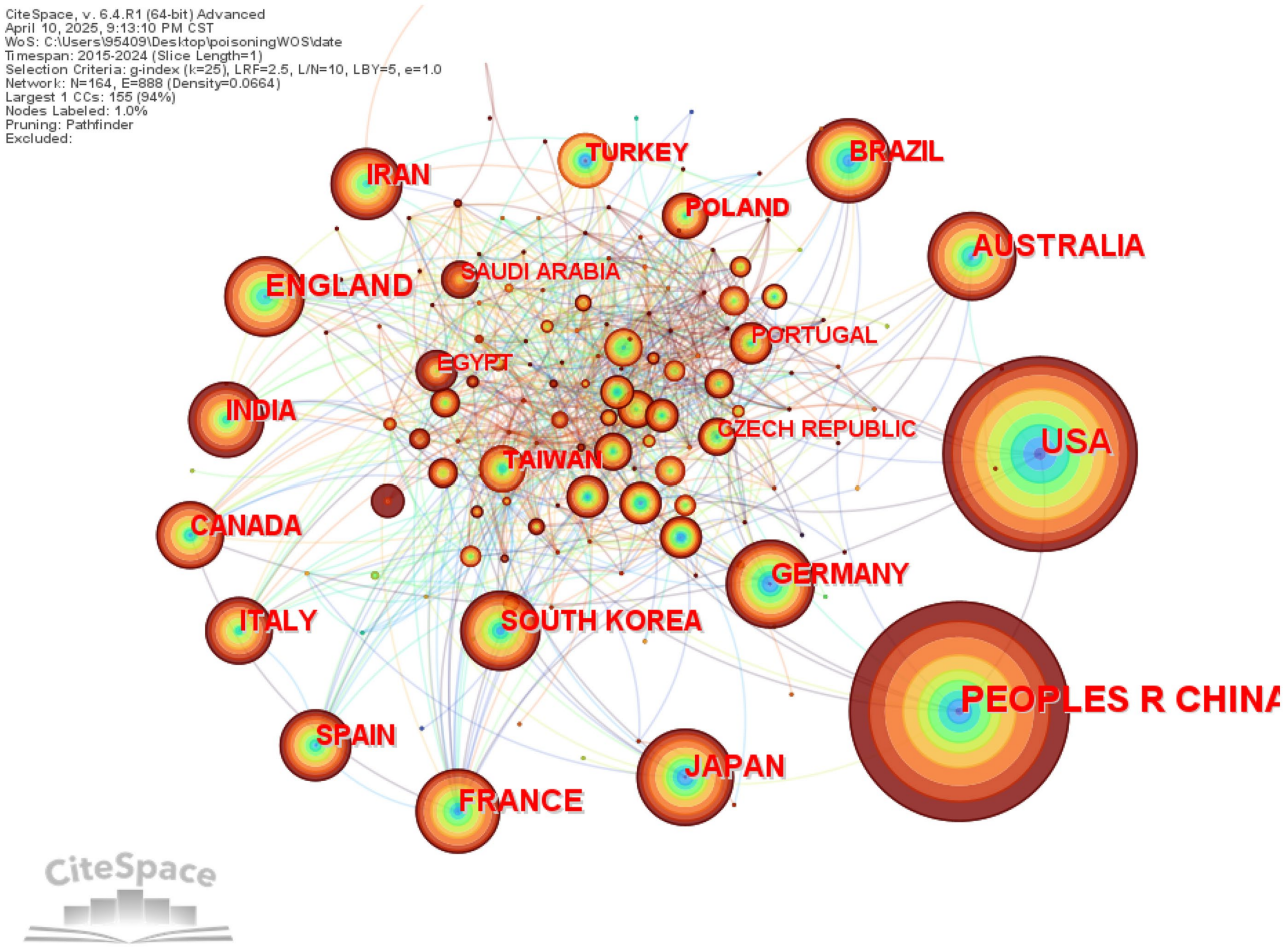
International collaboration network among countries/regions.

Through the visualized analysis of international collaboration networks (Figure 7), we can directly observe the characteristics of global scientific cooperation in this field. In the knowledge mapping representation, the size of each node (country) is proportional to its research output volume, while connecting lines indicate active research collaborations between nations. Notably, France, the United Kingdom, Spain and Canada have formed a relatively dense collaborative network with other countries, demonstrating particularly strong international research cooperation capabilities.

### 3.5 Keywords

#### 3.5.1 Co-occurrence analysis

Keywords in co-occurrence analysis can effectively reflect the research hotspots in a specific field (10). High-frequency and high-centrality keywords often indicate popular research topics within the domain. Excluding keywords related to the search terms such as “poisoning” “acute poisoning” and “toxicity” the high-frequency keywords in Chinese literature include “hemoperfusion” “nursing” “naloxone” “prognosis” and “paraquat” etc. In English literature high-frequency keywords include “exposure” “oxidative stress” “identification” “management” and “mortality” etc. In the Chinese literature keywords with a centrality > 0.1 indicate strong betweenness centrality and these keywords (“treatment” (centrality = 0.12) “blood purification” (centrality = 0.12) and “carbon monoxide” (centrality = 0.11)) represent research hotspots in the field (Table 4).

**Table 4:** Top 20 keywords in Chinese and English literature in the field of toxicology research.

Label	English literature			Chinese literature		
	Count	Centrality	Keywords	Count	Centrality	Keywords
1	1,138	0	toxicity	610	0.13	poisoning
2	989	0.01	exposure	496	0.17	acute poisoning
3	798	0.04	oxidative stress	470	0.05	hemoperfusion
4	547	0.01	identification	330	0.08	nursing
5	526	0.01	intoxication	274	0.04	naloxone
6	493	0.03	management	233	0.08	prognosis
7	439	0.01	mortality	225	0.09	paraquat
8	425	0.01	risk	173	0.07	respiratory failure
9	415	0.02	expression	173	0.07	hyperbaric oxygen
10	394	0.03	United States	166	0.05	first aid
11	353	0.02	prevalence	158	0.03	xingnaojing
12	326	0.03	health	157	0.05	atropine
13	326	0.01	children	153	0.09	acute
14	289	0.02	staphylococcus aureus	139	0.07	efficacy
15	282	0	carbon monoxide poisoning	118	0.12	treatment
16	278	0.02	overdose	115	0.04	hemodialysis
17	275	0.02	carbon monoxide	111	0.05	emergency
18	272	0.02	in vitro	110	0.12	blood purification
19	264	0.02	risk factors	107	0.04	clinical efficacy
20	258	0.01	inhibition	92	0.02	clinical effectiveness

**Figure 8:**
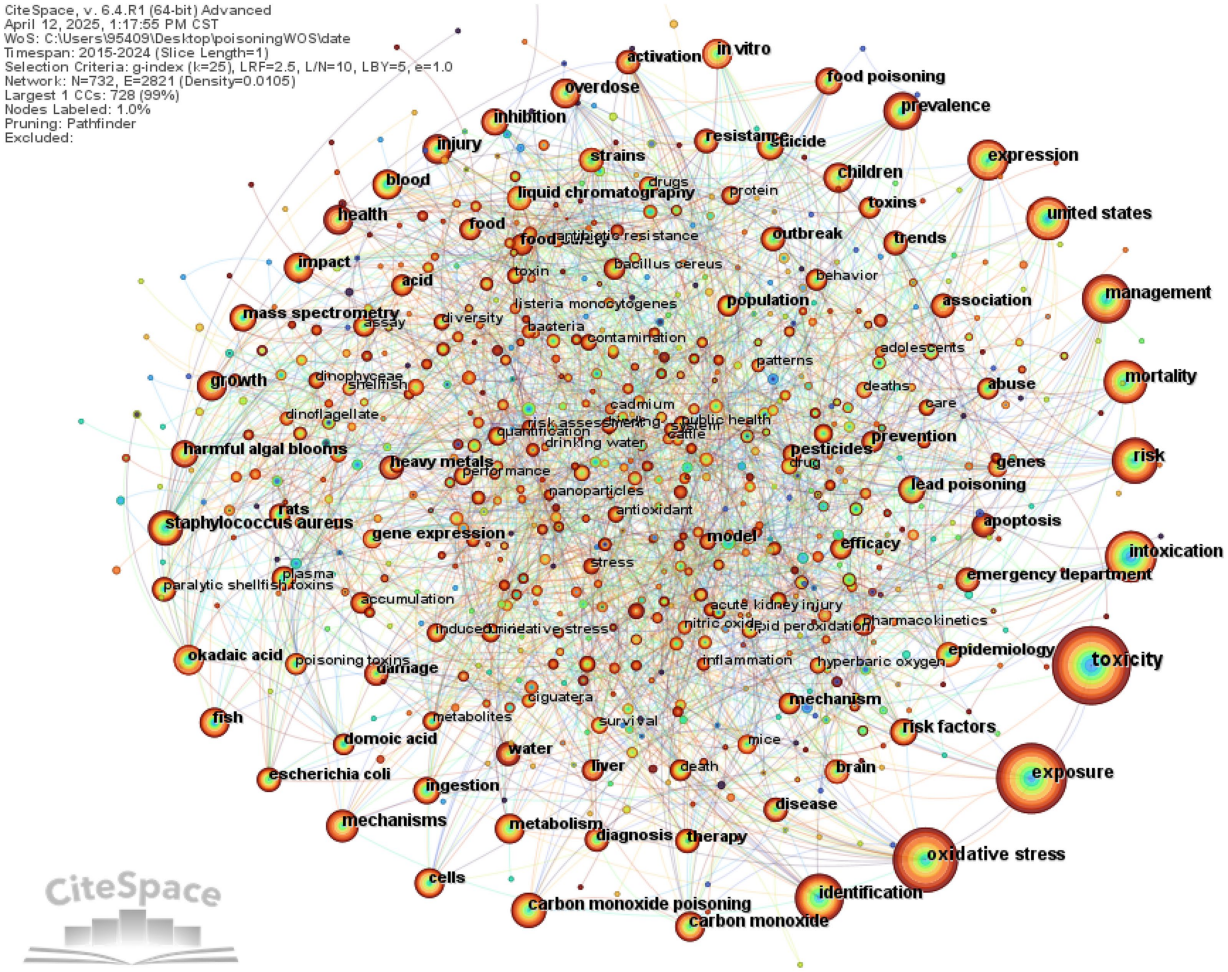
Keyword co-occurrence network in English-language poisoning research.

**Figure 9:**
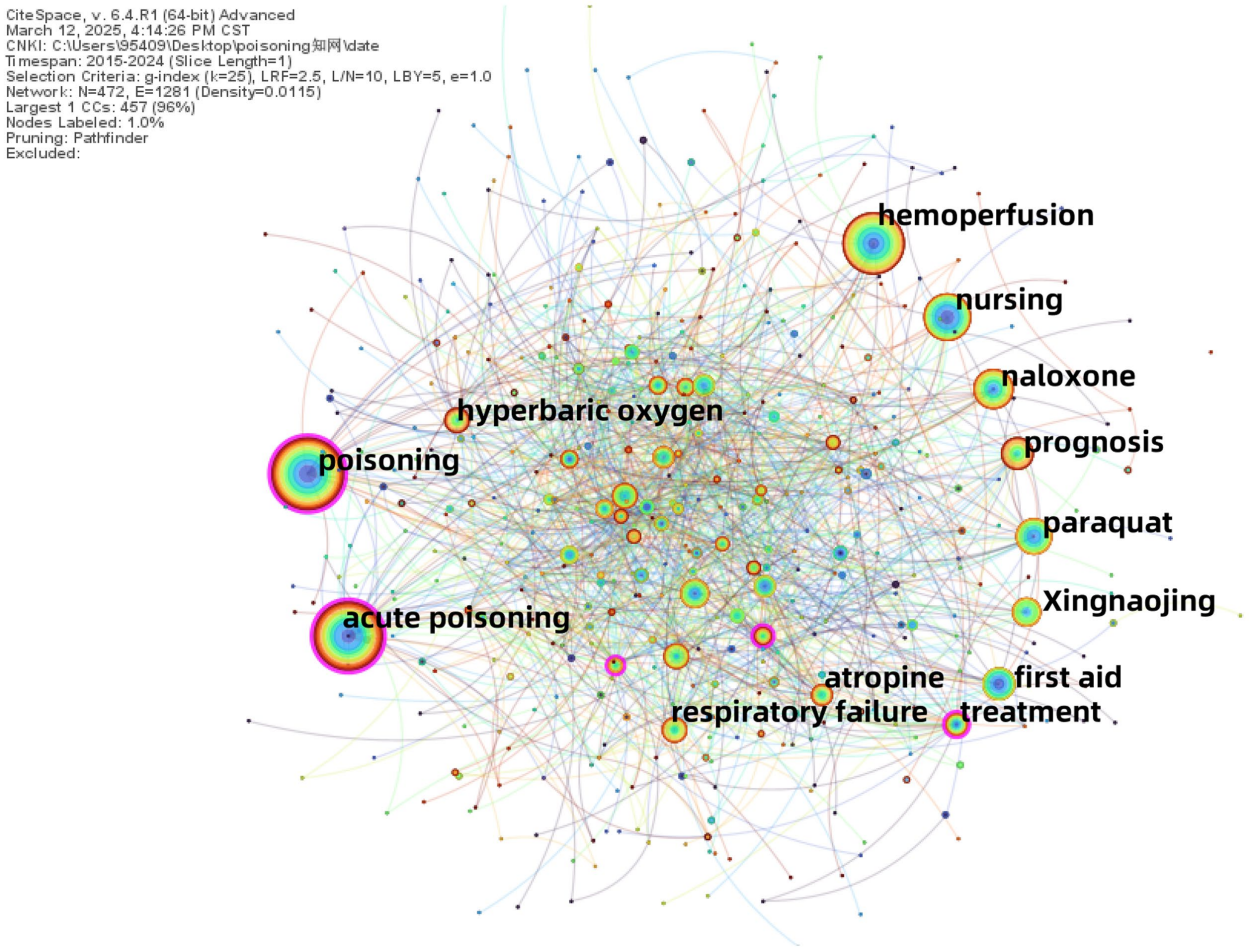
Keyword co-occurrence network in Chinese-language poisoning research.

Co-occurrence analysis maps were generated, where nodes represent keywords, node size indicates the frequency of keywords, different colors represent the years in which the keywords appeared, and connecting lines between nodes indicate relationships between keywords (Figures 8, 9).

#### 3.5.2 Cluster analysis

Keyword clustering involves grouping and clustering terms with similar or identical themes reflecting the composition of research topics within a field. The modularity value *Q* (>0.3 indicates a significant cluster structure) and the mean silhouette value *S* (>0.5 indicates reasonable clustering >0.7 indicates highly efficient and convincing clustering) were used as evaluation metrics. This study employed K-core clustering for analysis.

**Figure 10:**
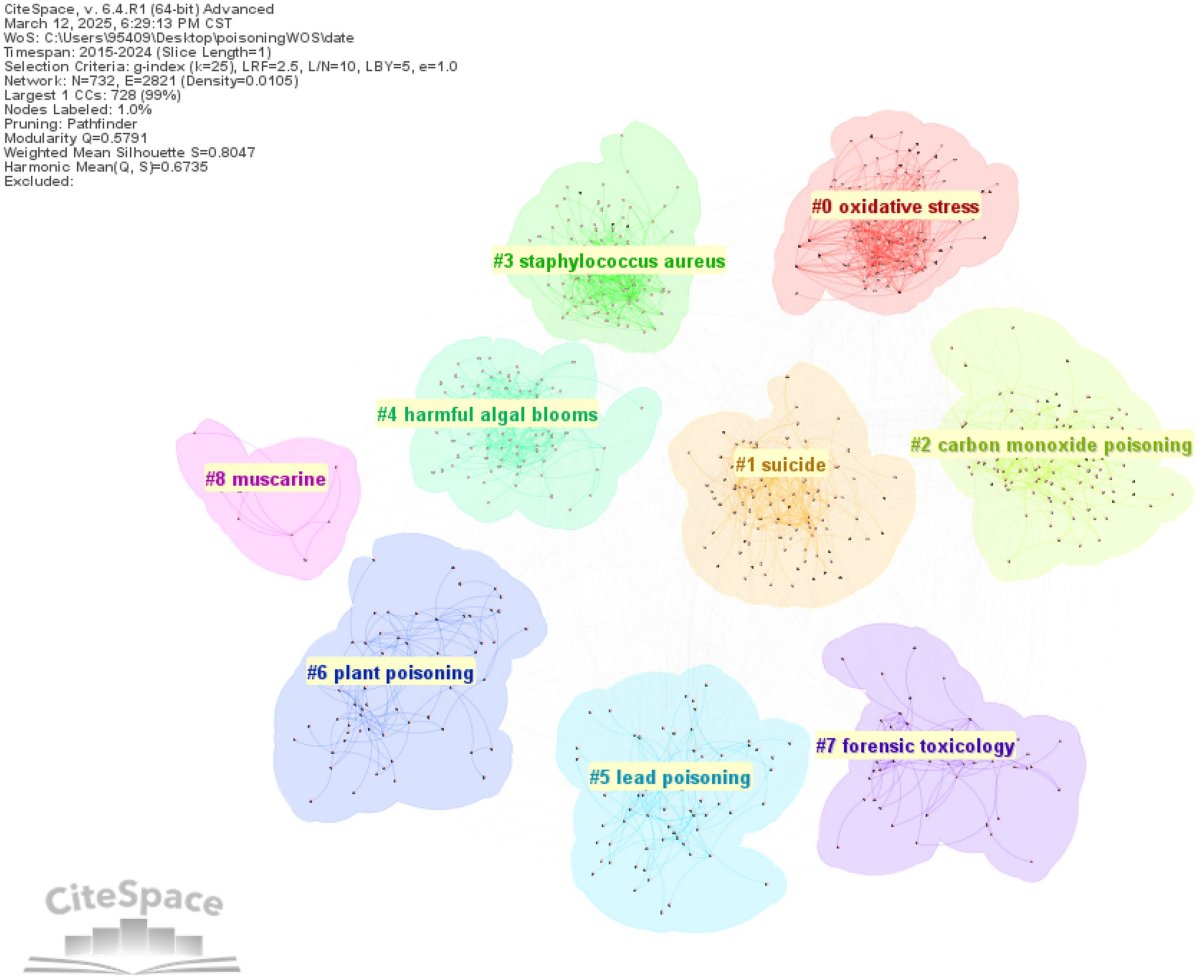
Keyword clustering network in English-language poisoning research.

For English literature, a total of 9 clusters were obtained, with *Q* = 0.5791 (>0.3) and *S* = 0.8047 (>0.7), indicating a significant and credible cluster structure. The clustering results were as follows: #0 oxidative stress, #1 suicide, #2 carbon monoxide poisoning, #3 staphylococcus, #4 harmful algal blooms, #5 lead poisoning, #6 plant poisoning, #7 forensic toxicology, and #8 muscarine (Figure 10).

**Figure 11:**
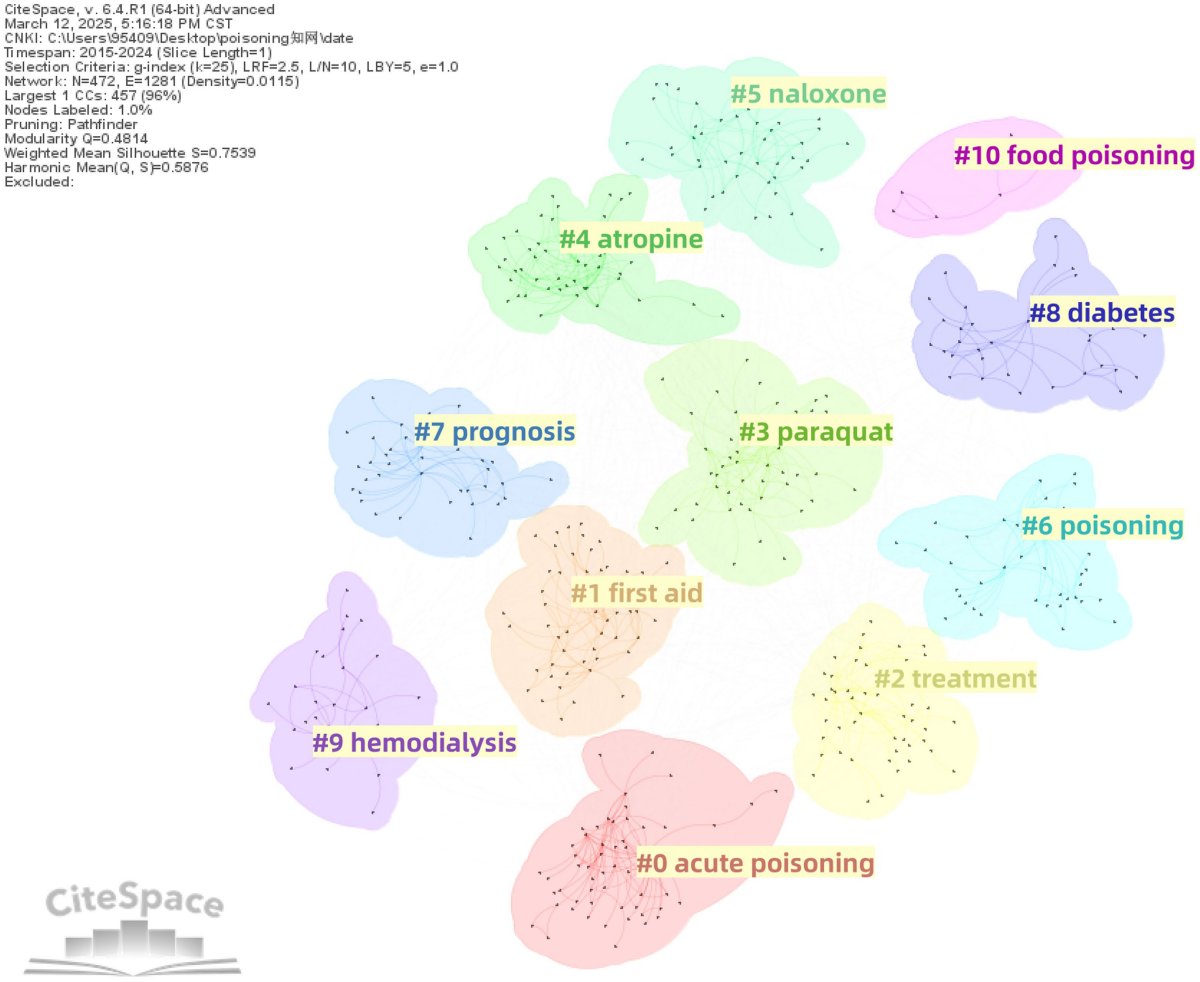
Thematic cluster network of keywords in Chinese poisoning research literature.

For Chinese literature, a total of 11 clusters were obtained, with *Q* = 0.4814 (>0.3) and *S* = 0.7539 (>0.7), indicating a significant and credible cluster structure. The clustering results were as follows: #0 acute poisoning, #1 first aid, #2 treatment, #3 paraquat, #4 atropine, #5 naloxone, #6 poisoning, #7 prognosis, #8 diabetes, #9 hemodialysis, and #10 food poisoning (Figure 11).

The emergence of these clusters can be attributed to research trends. For example, the forensic toxicology cluster (English #7) likely reflects the international academic focus on toxicological investigations in legal and criminal contexts, such as postmortem toxin analysis and drug-related fatalities. Similarly, the staphylococcus cluster (English #3) may represent studies on bacterial toxins (e.g., staphylococcal enterotoxins) and their role in foodborne illnesses. In contrast, Chinese literature places greater emphasis on clinical toxicology, particularly regarding acute poisoning (e.g., paraquat, #3) and antidote applications (e.g., atropine for organophosphate poisoning, #4; naloxone for opioid overdose, #5).

#### 3.5.3 Burst analysis

**Figure 12:**
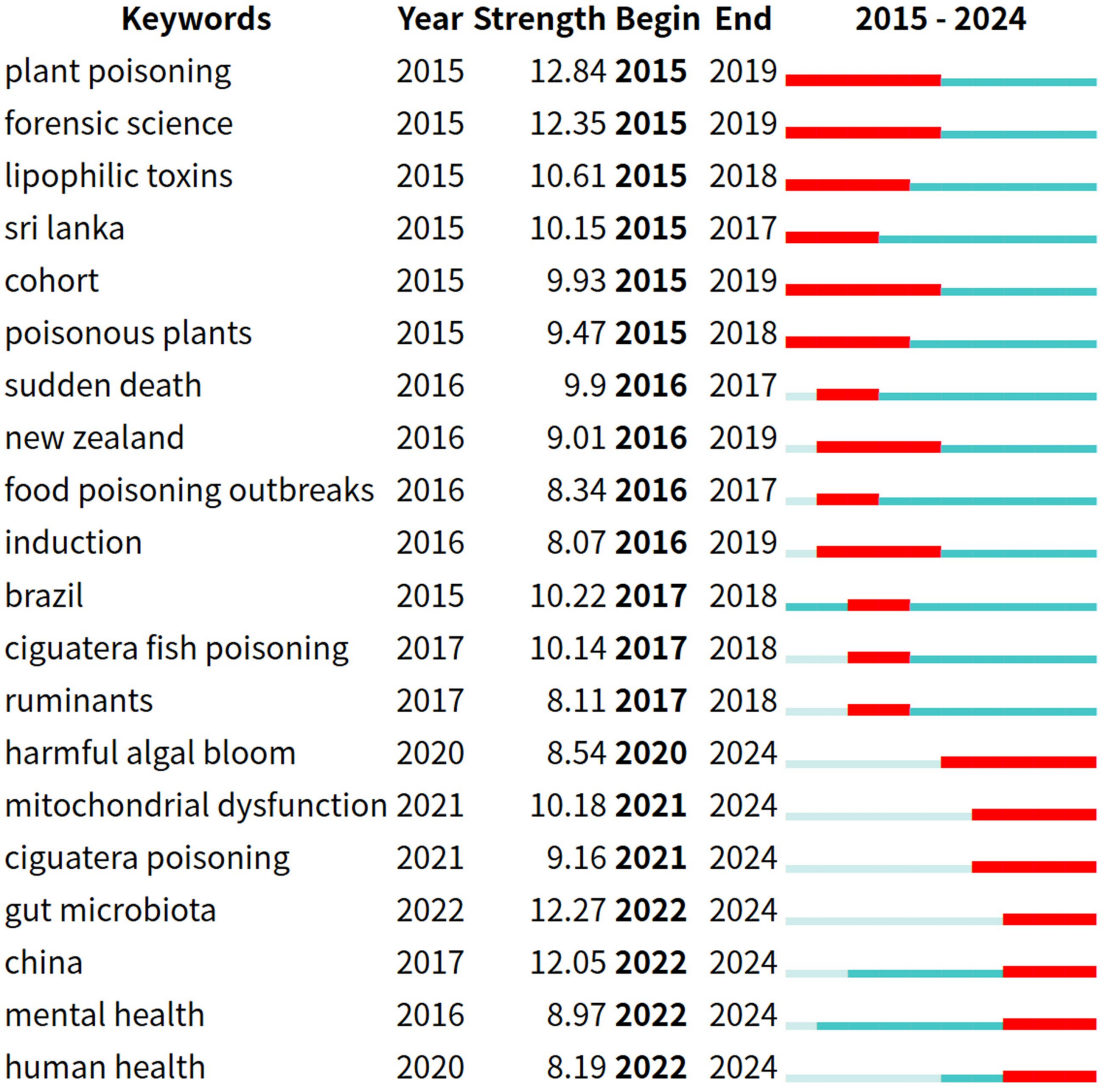
Top 20 burst keywords in English-language poisoning research literature.

**Figure 13:**
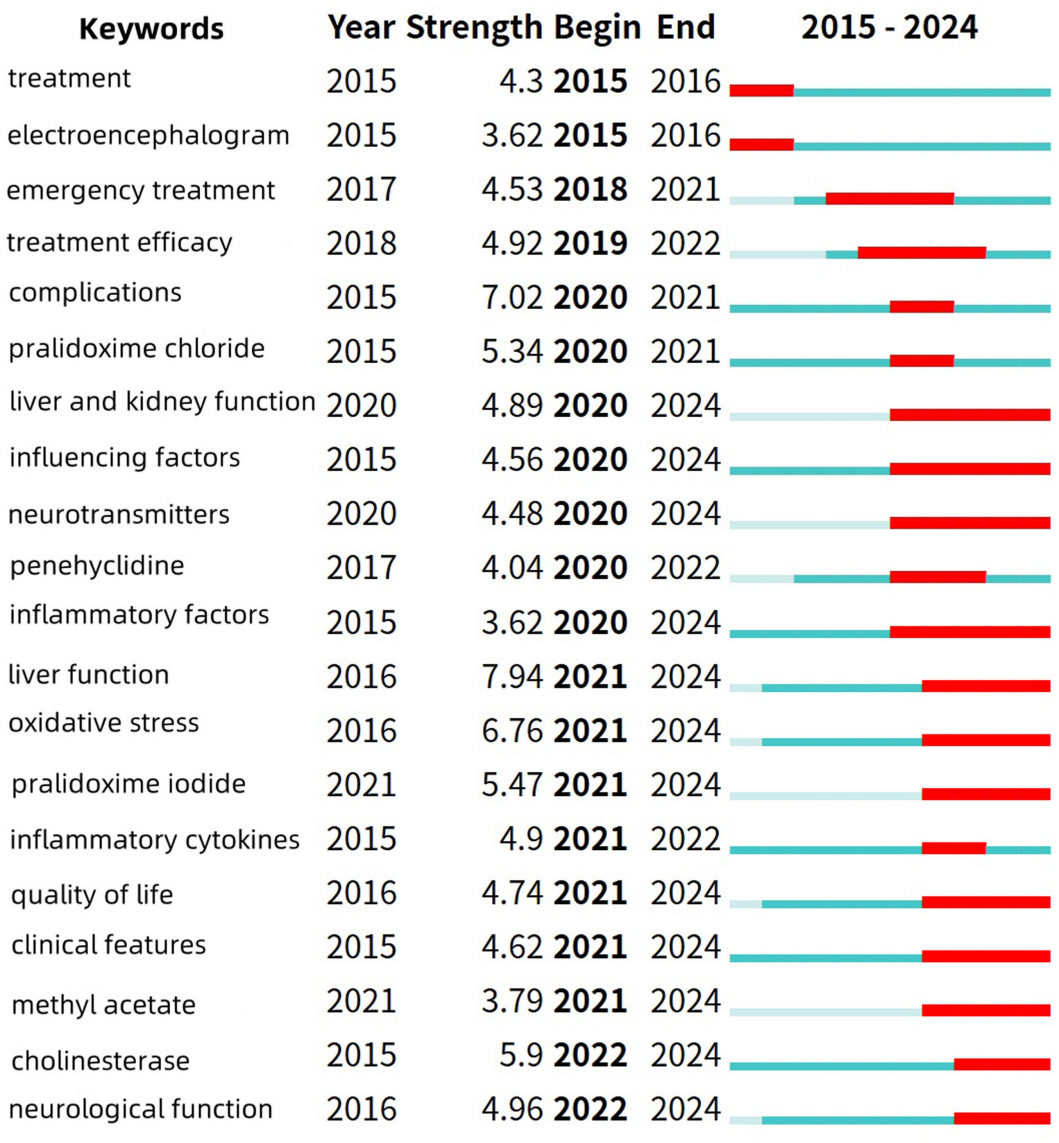
Top 20 burst keywords in Chinese poisoning research literature.

Burst terms refer to key terms that exhibit significant frequency fluctuations within specific time intervals, and their distribution characteristics can effectively reflect the dynamic evolution of academic research hotspots. In the keyword burst analysis map, the blue line represents the time interval, while the red line indicates the period during which the usage of the keyword significantly increased. “Year” denotes the earliest year the keyword appeared, “Strength” represents the burst strength, and “Begin” and “End” indicate the start and end times of the keyword’s burst, respectively (Figures 12, 13).

The top 20 burst terms were selected for both Chinese and English literature. Among the English literature, the terms with the highest burst strength include “plant poisoning,” “forensic science” and “gut microbiota.” The term that has recently become a hotspot is “gut microbiota.” In the Chinese literature, the terms with the highest burst strength are “liver function,” “complications” and “oxidative stress.” The terms that have recently become hotspots are “liver function” and “oxidative stress.”

## 4 Discussion

### 4.1 Shared research hotspots

Based on a comprehensive analysis of high-frequency keywords, high-betweenness centrality keywords, and cluster analysis, carbon monoxide (CO) emerges as a common research hotspot in both Chinese and English literature. This phenomenon is likely closely related to the prevalence and severity of carbon monoxide poisoning worldwide. CO is an inorganic compound gas that is colorless, odorless, and highly toxic. When inhaled in large quantities, CO rapidly binds to hemoglobin, disrupting the normal transport and supply of oxygen. This often leads to severe damage to the central nervous and cardiovascular systems, causing a range of pathological changes. Survivors of severe poisoning frequently suffer from significant neurological sequelae, such as mental abnormalities, Parkinson’s syndrome, or complications like delayed encephalopathy (11, 12).

### 4.2 Divergent research focuses

Although preventive interventions (such as public education and safety measures) have significantly reduced the incidence of poisoning, as a common clinical type of poisoning, the optimization of treatment protocols remains a critical issue to be addressed. Research trends indicate that innovative treatment strategies primarily focus on two directions: first, non-pharmacological interventions based on physical principles aimed at accelerating the dissociation of CO-hemoglobin complexes; and second, the development of portable emergency medications, particularly molecular agents with high-efficiency CO clearance capabilities, to enable immediate on-site treatment of poisoning (13).

English literature tends to focus more on epidemiological and molecular-level studies of poisoning. This inclination is reflected in high-frequency keywords such as “identification,” “prevalence,” “risk factors,” “exposure” and “oxidative stress.”

Firstly, in terms of epidemiological research, scholars have focused on the temporal and regional distribution patterns of poisoning incidents and population characteristics, such as in Egypt, Iran, and the United States. Each country has its unique research focus. For example, Egypt has conducted epidemiological surveys on acute poisoning among children visiting treatment centers (14), Iran has performed retrospective analyses of buprenorphine abuse (15), and the United States has conducted descriptive epidemiological analyses of occupational poisoning (16). The appearance of the high-frequency keyword “children” may reflect the international community’s heightened concern about poisoning incidents in vulnerable populations, particularly children, due to their physiological characteristics and sensitivity to environmental hazards (17, 18).

The in-depth analysis based on GBD 2019 data reveals significant geographical heterogeneity in the global burden of poisoning. Spatially, poisoning mortality rates in developing countries are 3.5 times higher than in high-income nations, with particularly striking disparities in Southeast Asia and Africa. These regions exhibit alarmingly high age-standardized mortality rates (ASMR) of 4.2 per 100,000 population from pesticide poisoning (e.g., organophosphates), substantially exceeding the global average of 1.8 per 100,000. These regional disparities primarily stem from gaps in the enforcement of agricultural chemical regulations, accessibility of primary emergency medical resources, and the reach of public health education programs. The GBD data identifies two key high-risk population groups: among 15–49 year-olds, suicide-related poisonings (e.g., paraquat, opioids) account for 42% of all poisoning deaths, while in low-income countries, accidental poisonings (e.g., ingestion of household cleaners or medications) constitute 28% of cases among children under five. These epidemiological patterns are corroborated by our keyword co-occurrence analysis, where high-frequency terms such as “exposure” and “risk factors” in English-language literature show strong alignment with the exposure patterns revealed in the GBD study (2).

Secondly, at the molecular level, English literature emphasizes the role of various toxins in inducing oxidative stress or the protective effects of antidotes through oxidative stress modulation. For instance, acute dichlorvos (DDVP) poisoning can significantly elevate blood glucose levels in broiler chickens, accompanied by an imbalance in redox homeostasis (19). The core toxic mechanism of acute CO poisoning stems from the excessive generation of reactive oxygen species (ROS) and reactive nitrogen species (RNS), leading to the collapse of the body’s antioxidant defense system (20). Additionally, in studies on methylmercury (MeHg) toxicity, ghrelin has been found to potentially exert neuroprotective effects by regulating oxidative stress pathways (21). Lastly, infections caused by Staphylococcus species, such as *Staphylococcus aureus*, are also a research hotspot.

Chinese literature primarily focuses on clinical treatment and prognosis in poisoning research. This is reflected in high-frequency keywords such as “hemoperfusion,” “nursing” and “prognosis” as well as high-betweenness centrality keywords like “treatment” and “blood purification,” along with cluster analysis results.

Blood purification plays a central role in poisoning treatment and is a critical method for managing severe poisoning cases. Hemoperfusion (HP) is a technique that involves circulating blood through an external adsorption device to directly remove toxins or metabolites using adsorbents such as activated charcoal or resin. It is particularly effective for toxins with high lipid solubility and protein binding rates, such as organophosphorus pesticides. HP can rapidly reduce toxin concentrations in the blood, mitigating damage to target organs. It is used for various drug poisonings (e.g., barbiturates, theophylline) and chemical poisonings (e.g., paraquat) (22–24).

The high-frequency keywords “prognosis” and “nursing” reflect the “patient-centered” service philosophy of the Chinese healthcare system. This characteristic highlights China’s focus not only on acute-phase rescue and treatment but also on long-term rehabilitation and quality-of-life improvement for patients. It also indicates a shift in China’s poisoning treatment field from solely treatment to comprehensive management.

### 4.3 Future research trajectories

The burst analysis results of Chinese literature indicate that liver function and oxidative stress are likely to become key research directions in China in the future.

It is noteworthy that these two research directions–liver function and oxidative stress–are closely interconnected. As the primary metabolic organ, the liver plays a crucial role in the occurrence and development of oxidative stress. Simultaneously, oxidative stress is one of the key mechanisms leading to liver function damage. Therefore, future research in China may increasingly focus on the intersection of these two areas.

The keyword burst analysis results for English literature reveal that the term with the highest burst strength over the past 3 years and ongoing research interest is “gut microbiota.” This suggests that gut microbiota may become an emerging research direction and hotspot in the field of poisoning research. The gut microbiota refers to the complex microbial community residing in the human digestive tract, comprising up to 10^14 microorganisms and over 1,000 different bacterial species, playing a vital role in maintaining human health. In poisoning research, the gut microbiota has demonstrated unique detoxification potential. For example, in heavy metal poisoning, Luo et al. found that microbial detoxification shows promise in treating subchronic arsenic poisoning (25). Wang et al. through animal experiments, discovered that intervening in the gut microbiota composition of manganese-exposed rats significantly improved their neuroinflammatory responses, opening new therapeutic avenues based on gut microbiota remodeling for manganese poisoning treatment (26).

However, current research on the role of gut microbiota in poisoning still faces several challenges. The specific molecular mechanisms by which the gut microbiota participates in detoxification remain incompletely understood. Although studies have shown that the gut microbiota can exert detoxification effects through metabolic transformation, competitive binding, immune regulation, and other pathways, the signaling pathways and key molecules involved in these processes require further clarification (27). Additionally, the long-term safety and efficacy of gut microbiota-based interventions, such as probiotic supplementation (28) and fecal microbiota transplantation (29), need more clinical data to support their application.

### 4.4 Strengths and limitations

This study represents the first global bibliometric analysis of poisoning research employing CiteSpace visualization technology. By systematically collecting high-quality literature data from both the Web of Science and CNKI databases, we ensured sufficient sample size and representativeness, thereby enhancing the scientific validity and reliability of our findings. However, it should be noted that the exclusion of databases such as PubMed and Wanfang may limit coverage of certain regional or specialized research outputs. Future studies could expand data sources and employ cross-validation across multiple databases to further improve the comprehensiveness and accuracy of research conclusions.

## 5 Conclusion

In recent years, research related to poisoning has shown an overall upward trend. Currently, the main research hotspots in this field include epidemiology, molecular-level studies (such as oxidative stress), clinical treatment (e.g., blood purification), prognosis, and chemical substances (e.g., CO, paraquat). Research on gut microbiota in the field of poisoning may become a new breakthrough, as its role in toxin metabolism, detoxification mechanisms, and individual susceptibility holds promise for providing new insights into the prevention and treatment of poisoning. Meanwhile, studies on the molecular mechanisms of oxidative stress will continue to deepen, potentially revealing new therapeutic targets in the future.
