# Supplementary material for: Visualization analysis of poisoning-related research based on CiteSpace
Source: Front Public Health. 2025 May 7;13:1592916. doi: 10.3389/fpubh.2025.1592916 (PMC12092380; doi:10.3389/fpubh.2025.1592916)
Supplement: Supplementary file 1 [file Data_Sheet_1.PDF]

1.Since the frequency of carbon monoxide ranks 28th in Chinese literature, the text only lists the top 20 high-frequency keywords, but the intermediary centrality of carbon monoxide keywords needs to be used in the text, so it is submitted as supplementary materials. （Figure 1）

Visi...	Count	Centrality	Year	Keywords
✓	610	0.13	2015	中毒
✓	496	0.17	2015	急性中毒
✓	470	0.05	2015	血液灌流
✓	330	0.08	2015	护理
✓	274	0.04	2015	纳洛酮
✓	233	0.08	2015	预后
✓	225	0.09	2015	百草枯
✓	173	0.07	2015	呼吸衰竭
✓	173	0.07	2015	高压氧
✓	166	0.05	2015	急救
✓	158	0.03	2015	醒脑静
✓	157	0.05	2015	阿托品
✓	153	0.09	2015	急性
✓	139	0.07	2015	疗效
✓	118	0.12	2015	治疗
✓	115	0.04	2015	血液透析
✓	111	0.05	2015	急诊
✓	110	0.12	2015	血液净化
✓	107	0.04	2015	临床疗效
✓	92	0.02	2015	临床效果
✓	92	0.04	2015	心肌损伤
✓	90	0.07	2015	并发症
✓	85	0.04	2015	效果
✓	80	0.03	2015	抢救
✓	78	0.04	2015	洗胃
✓	77	0.06	2015	院前急救
✓	74	0.04	2015	儿童
✓	73	0.11	2015	一氧化碳
✓	71	0.06	[illegible]	[illegible]
✓	67	0.05	[illegible]	[illegible]

carbon monoxide

2.Since the visual analysis results generated by CiteSpace software for Chinese literature are in Chinese, Photoshop (PS) was used to translate the Chinese text in the figures into English. The original figures are provided below: （Figure 2，3，4）

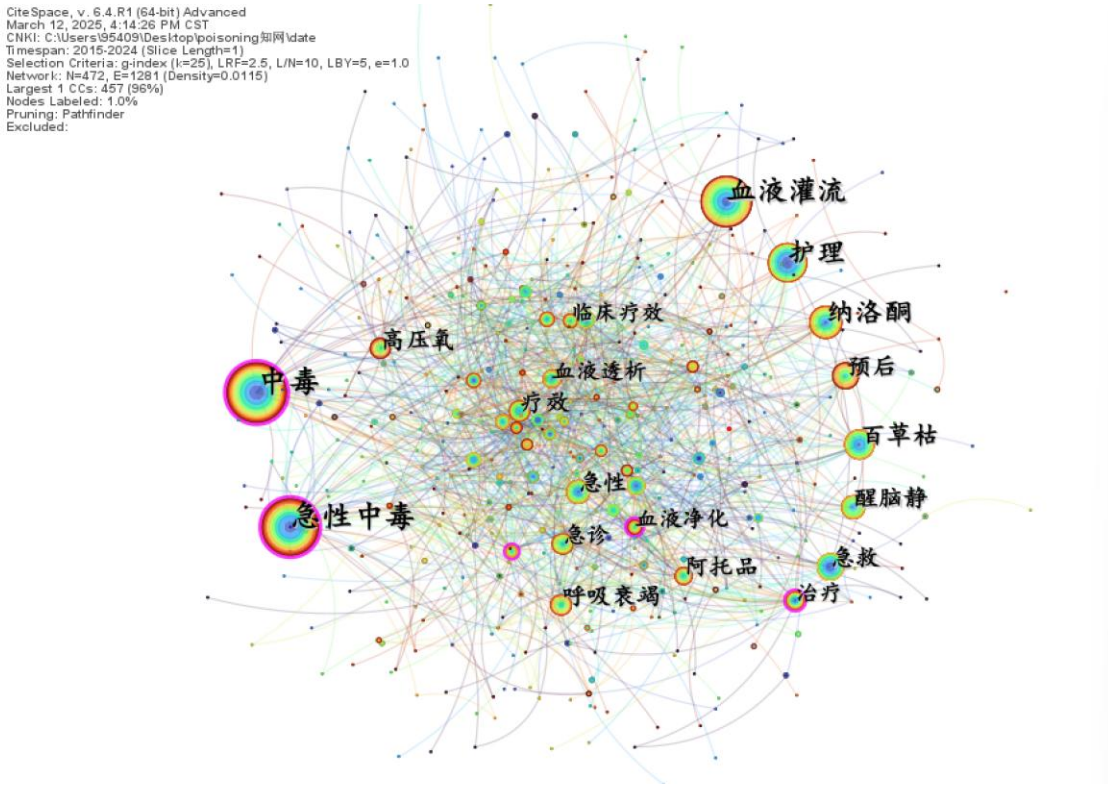

Figure 2　Keyword Co-occurrence Analysis of Chinese Literature

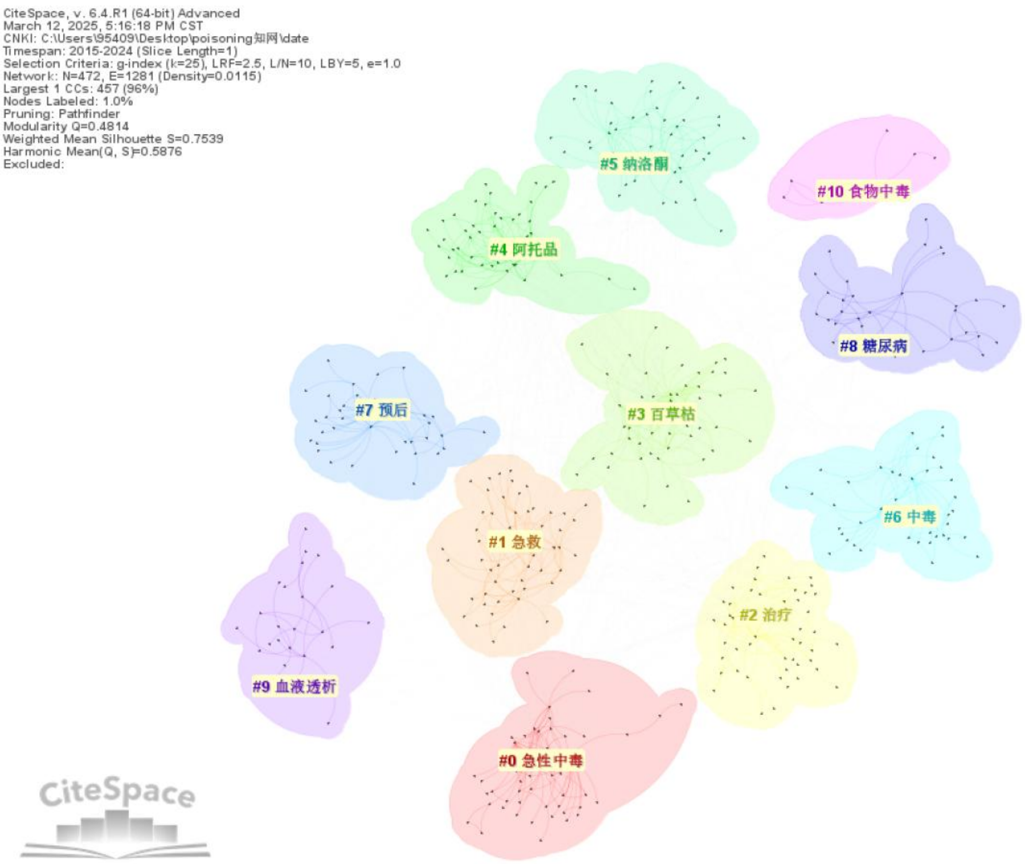

Figure 3　Keyword Cluster Analysis of Chinese Literature

Keywords	Year	Strength	Begin	End
救治	2015	4.3	**2015**	2016
脑电图	2015	3.62	**2015**	2016
急诊治疗	2017	4.53	**2018**	2021
救治效果	2018	4.92	**2019**	2022
并发症	2015	7.02	**2020**	2021
氯解磷定	2015	5.34	**2020**	2021
肝肾功能	2020	4.89	**2020**	2024
影响因素	2015	4.56	**2020**	2024
神经递质	2020	4.48	**2020**	2024
戊乙奎醚	2017	4.04	**2020**	2022
炎性因子	2015	3.62	**2020**	2024
肝功能	2016	7.94	**2021**	2024
氧化应激	2016	6.76	**2021**	2024
碘解磷定	2021	5.47	**2021**	2024
炎症因子	2015	4.9	**2021**	2022
生活质量	2016	4.74	**2021**	2024
临床特征	2015	4.62	**2021**	2024
乙酸甲酯	2021	3.79	**2021**	2024
胆碱酯酶	2015	5.9	**2022**	2024
神经功能	2016	4.96	**2022**	2024

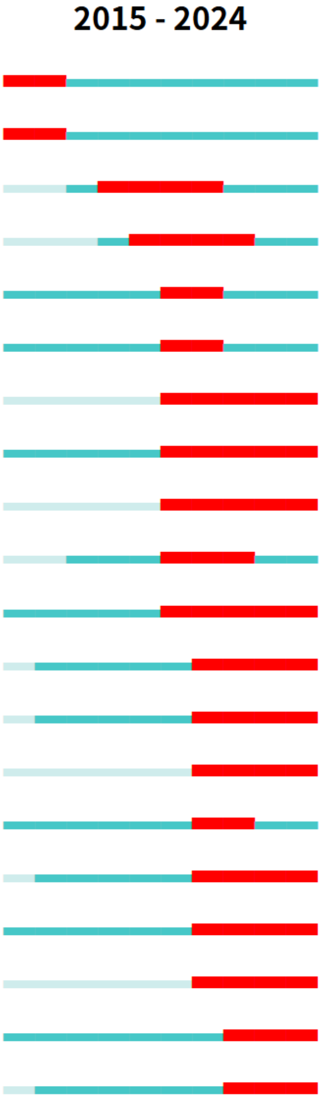

Figure 4 Keyword Burst Analysis of Chinese Literature
